# An October Parade at Gravesend

**Published:** 1911-09-30

**Authors:** 


### An October Parade at Gravesend.

The Parade and Carnival organised by the working-men of the district, which was held in aid of the funds of the Hospital in July, had to be abandoned owing to a severe thunderstorm. The Committee are anxious that the amount finally handed over shall equal that of previous years, and in order to make up the deficiency they have arranged another parade for Saturday, October 7.

